# High-Surety Isothermal Amplification and Detection of SARS-CoV-2

**DOI:** 10.1128/mSphere.00911-20

**Published:** 2021-05-19

**Authors:** Sanchita Bhadra, Timothy E. Riedel, Simren Lakhotia, Nicholas D. Tran, Andrew D. Ellington

**Affiliations:** a Department of Molecular Biosciences, College of Natural Sciences, The University of Texas at Austin, Austin, Texas, USA; b Center for Systems and Synthetic Biology, The University of Texas at Austin, Austin, Texas, USA; c Freshman Research Initiative, DIY Diagnostics Stream, The University of Texas at Austin, Austin, Texas, USA; Georgetown University

**Keywords:** loop-mediated isothermal amplification, strand displacement probes, SARS-CoV-2, multiplex assays, isothermal nucleic acid diagnostics, lateral flow assays, Boolean logic

## Abstract

Isothermal nucleic acid amplification tests (iNATs), such as loop-mediated isothermal amplification (LAMP), are good alternatives to PCR-based amplification assays, especially for point-of-care and low-resource use, in part because they can be carried out with relatively simple instrumentation. However, iNATs can often generate spurious amplicons, especially in the absence of target sequences, resulting in false-positive results. This is especially true if signals are based on non-sequence-specific probes, such as intercalating dyes or pH changes. In addition, pathogens often prove to be moving, evolving targets and can accumulate mutations that will lead to inefficient primer binding and thus false-negative results. Multiplex assays targeting different regions of the analyte and logical signal readout using sequence-specific probes can help to reduce both false negatives and false positives. Here, we describe rapid conversion of three previously described SARS-CoV-2 LAMP assays that relied on a non-sequence-specific readout into individual and multiplex one-pot assays that can be visually read using sequence-specific oligonucleotide strand exchange (OSD) probes. We describe both fluorescence-based and Boolean logic-gated colorimetric lateral flow readout methods and demonstrate detection of SARS-CoV-2 virions in crude human saliva.

**IMPORTANCE** One of the key approaches to treatment and control of infectious diseases, such as COVID-19, is accurate and rapid diagnostics that is widely deployable in a timely and scalable manner. To achieve this, it is essential to go beyond the traditional gold standard of quantitative PCR (qPCR) that is often faced with difficulties in scaling due to the complexity of infrastructure and human resource requirements. Isothermal nucleic acid amplification methods, such as loop-mediated isothermal amplification (LAMP), have been long pursued as ideal, low-tech alternatives for rapid, portable testing. However, isothermal approaches often suffer from false signals due to employment of nonspecific readout methods. We describe general principles for rapidly converting nonspecifically read LAMP assays into assays that are read in a sequence-specific manner by using oligonucleotide strand displacement (OSD) probes. We also demonstrate that inclusion of OSD probes in LAMP assays maintains the simplicity of one-pot assays and a visual yes/no readout by using fluorescence or colorimetric lateral-flow dipsticks while providing accurate sequence-specific readout and the ability to logically query multiplex amplicons for redundancy or copresence. These principles not only yielded high-surety isothermal assays for SARS-CoV-2 but might also aid in the design of more sophisticated molecular assays for other analytes.

## INTRODUCTION

**FIG 1:**
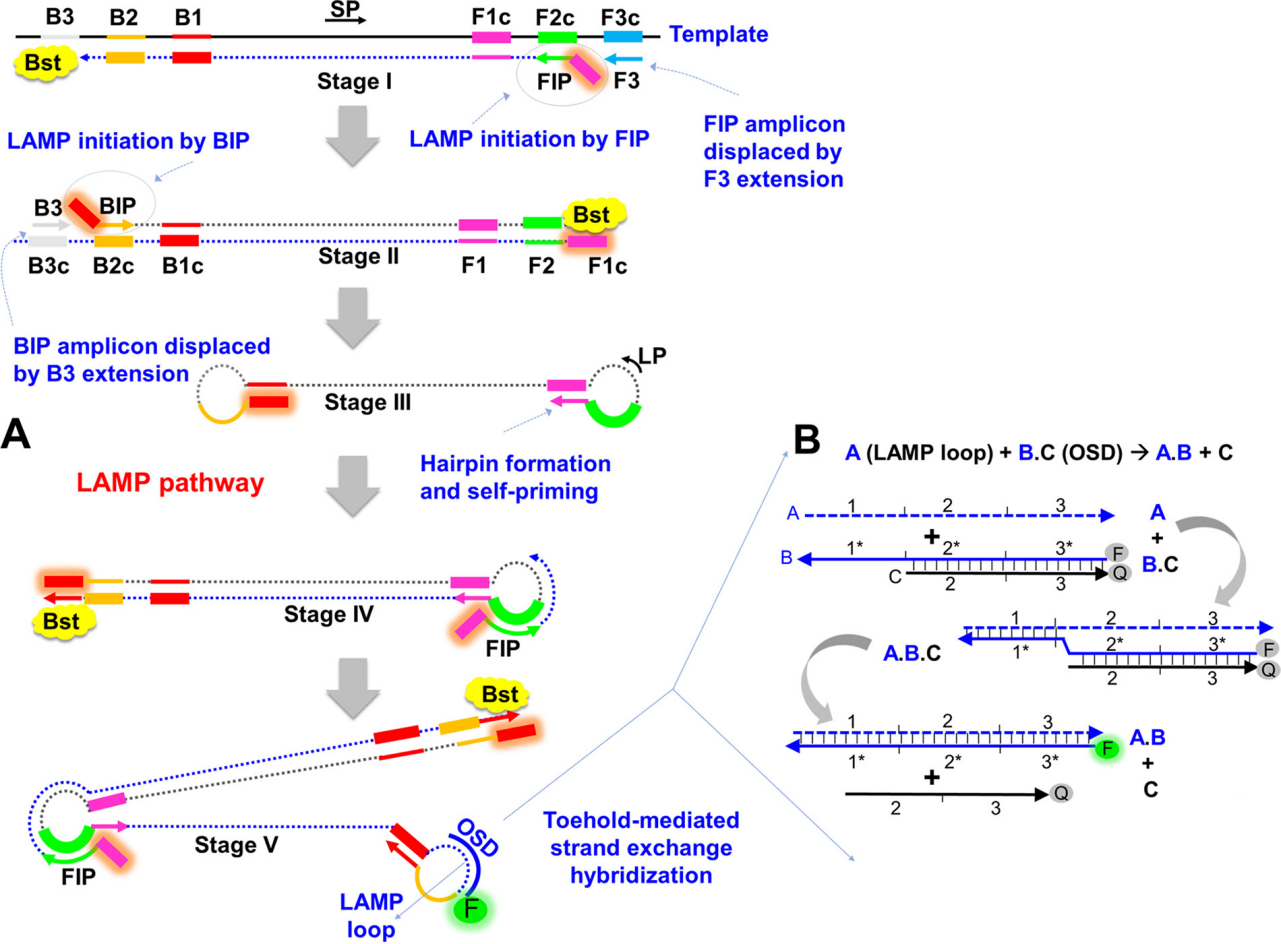
LAMP-OSD schematic. (A) Schematic depicting LAMP mechanism, where FIP and BIP indicate inner primers, B3 and F3 indicate outer primers, SP indicates stem primers, LP indicates loop primers, *Bst* indicates strand-displacing DNA polymerase, and c denotes complementary sequences. (B) Schematic depicting OSD design and toehold-mediated strand exchange process, where the strand labeled A represents the LAMP loop sequence and the B.C complex represents the hemiduplex OSD probe. F and Q on the OSD denote fluorophore and quencher, respectively. OSD and subsequent strand exchange intermediates are denoted by numbered domains, which represent short (usually <12-nucleotide) sequences in an otherwise continuous oligonucleotide. Complementary domains are indicated by asterisks.

Loop-mediated isothermal amplification (LAMP) uses the strand-displacing *Bst* DNA polymerase and four primers (FIP, BIP, F3, and B3) that bind to six target regions (B3, B2, B1, F1c, F2c, and F3c) to generate 10^9^ to 10^10^ copies of DNA or RNA targets, typically within 1 to 2 h (Fig. 1) (1). In greater detail, F2 in FIP (F1c-F2) and B2 in BIP (B1c-B2) initiate amplification. F1c and B1c self-prime subsequent amplification. F3- and B3-initiated DNA synthesis displaces FIP- and BIP-initiated strands. 3′ ends of the resulting single-stranded, dumbbell-shaped amplicons are extended to hairpins by *Bst* polymerase. FIP and BIP hybridize to the single-stranded loops and initiate DNA synthesis that opens the hairpin to form concatameric amplicons containing self-priming 3′-end hairpins. The ensuing continuous amplification generates double-stranded concatameric amplicons with self-priming hairpins and single-stranded loops (1).

LAMP can rival PCR for sensitivity without thermocycling (2), and additional stem and loop primers (LB and LF for backward and forward loop primers, respectively) can accelerate amplification, with some LAMP assays being complete within 10 min (3, 4). However, since LAMP is commonly read by using nonspecific methods (such as Mg^2+^ precipitation, intercalating dyes, or labeled primers) that cannot distinguish spurious amplicons that frequently arise from continuous amplification, its utility can be limited. We have previously overcome these drawbacks by using oligonucleotide strand exchange (OSD) probes (5), based in part on advances in strand exchange DNA computation (Fig. 1) (6). Strand exchange occurs when two partially or fully complementary strands hybridize to each other by displacing a prehybridized strand(s) (Fig. 1B). Strand exchange usually initiates by base pairing at single-stranded “toeholds” and progresses to form additional base pairs via branch migration, allowing the rational design of complex algorithms and programmable nanostructures (7–11). The hemiduplex OSD probes contain a so-called “toehold” that allows sequence-specific interaction with a target molecule and have opposed fluorophore and quencher moieties. In the presence of a complementary target, the OSD probes can undergo strand exchange and separation, leading to an easily read fluorescence signal (5). In essence, the OSD probes are functional equivalents of TaqMan probes and have been shown to accurately report single or multiplex LAMP amplicons from few tens of targets without interference from nonspecific amplicons or inhibitors (5, 12). Of equal importance, the programmability of OSD probes allows their adaptation to many different assay formats, including readout of LAMP signal using off-the-shelf devices such as glucometers and colorimetric lateral flow dipsticks for pregnancy hormones or fluorescein (13–18).

LAMP-OSD is designed consciously to be easy to use and interpret, which makes it a reliable choice for either screening or validation of disease states. Base pairing to the toehold region is extremely sensitive to mismatches, ensuring specificity, and the programmability of both primers and probes makes possible rapid adaptation to new diseases or new disease variants. We have shown that higher-order molecular information processing is also possible, such as integration of signals from multiple amplicons (19). Overall, the use of sequence-specific probes allows construction of strand exchange computation circuits that act as “matter computers” (7–10), something that is not generally possible within the context of a PCR (which would of necessity melt the computational devices).

We have taken pains to make LAMP-OSD robust for resource-poor settings. Lyophilized master mixes are stable without cold chain for extended durations and can be operated simply upon rehydration and addition of crude sample (20). The one-pot operation, direct analysis of crude specimens, and easy yes/no visual readout make LAMP-OSD ideal for field operation with minimal training and resources.

OSD probes can be readily designed for integration into existing LAMP assays without significant disruption to standard assay practice. To that end, here we demonstrate the conversion of three recently described LAMP primer sets for detection of SARS-CoV-2 but that use nonspecific readout methods (see Table S1 in the supplemental material). The individual and multiplexed LAMP-OSD versions of these assays maintain the simplicity of a visual yes/no readout while endowing the assays with the inherent accuracy of probe-based signal transduction, including conversion to Boolean AND logic-gated colorimetric readout of amplicon copresence on lateral flow dipsticks. We also demonstrate the feasibility of sample-to-answer operation of LAMP-OSD by directly analyzing human saliva spiked with SARS-CoV-2 virions.

10.1128/mSphere.00911-20.2 TABLE S1 Prepublished LAMP primer sets for SARS-CoV-2 found online before 4 March 2020. Download Table S1, DOCX file, 0.02 MB. Copyright © 2021 Bhadra et al. 2021 Bhadra et al. https://creativecommons.org/licenses/by/4.0/ This content is distributed under the terms of the Creative Commons Attribution 4.0 International license.

(An earlier version of this article was submitted to an online preprint archive [21]).

## RESULTS

### Integration of OSD probes into prepublished SARS-CoV-2 LAMP primer sets.

A series of 11 recently described primer sets for SARS-CoV-2 was screened using WarmStart colorimetric LAMP 2X master mix (NEB, Ipswich, MA, USA) according to the manufacturer’s protocol (see Table S1 in the supplemental material). Spurious amplification was also assessed in standard real-time reverse transcription (RT)-LAMP reactions assembled from individual components where OSD reporters were replaced with the intercalating fluorophore EvaGreen (Biotium, Hayward, CA, USA), according to the manufacturer’s instructions. While there was some variation in the false-positive rates between the two assay methods, likely due to differences in assay composition and readout sensitivity, we found overall that 9 of the 11 sets showed significant no-template amplification often in more than 10% of the replicates in less than an hour of incubation at 63 to 65°C (Fig. S2). These results are consistent with other published results that rely on nonspecific readout, such as colorimetric LAMP reactions, rather than on nucleic acid probe-based detection (22). In fact, for many published assays, color changes must be read within a narrow window of time in order to minimize spurious conclusions, a consideration that does not scale well for diagnostic screening, especially at point of care or as an early part of a clinical diagnostics pipeline.

10.1128/mSphere.00911-20.4 FIG S2 Nonspecific amplification profile of a set of (pre)published SARS-CoV-2 LAMP primers. Primer sets listed in Table S1 were tested for nonspecific amplification at preprint amplification temperatures indicated in Table S1 using 10 replicate reactions each of real-time EvaGreen RT-LAMP and colorimetric pH LAMP that did not receive any viral templates. Amplification curves generated by measuring EvaGreen fluorescence in real time are depicted in the top panel for each primer set. Images of colorimetric LAMP reaction color taken after 60 min of amplification are shown in the bottom panel for each primer set. False-positive color reactions are circled in blue. Data are representative of two biological replicates. Download FIG S2, TIF file, 1.3 MB. Copyright © 2021 Bhadra et al. 2021 Bhadra et al. https://creativecommons.org/licenses/by/4.0/ This content is distributed under the terms of the Creative Commons Attribution 4.0 International license.

To suppress potential false-positive readouts, we chose to develop OSD probes for three of the LAMP primer sets, here termed NB, Lamb, and Tholoth (Table 1 and Fig. S1). These primer sets target three different regions of the viral genome, the N gene, the NSP3 coding region of ORF1AB, and the RNA-dependent RNA polymerase coding region of ORF1AB. Of the three primer sets, the NB assay had the lowest propensity for spurious signal when analyzed by nonspecific colorimetric readout or by fluorescence dye-based measurements (Fig. S2). Similarly, the Lamb primer set displayed minimal nonspecific amplification. However, the Tholoth assay demonstrated a frequent tendency for false signals. To create LAMP-OSD versions of these assays, we designed OSD probes that were complementary to one of the loop sequences in each of the three LAMP amplicons. Subsequently, Tholoth, Lamb, and NB LAMP-OSD assays were set up individually by mixing separate reaction components as indicated in Materials and Methods. Each individual assay contained its specific OSD probes along with both inner primers, FIP and BIP, and both outer primers, F3 and B3. In addition, each assay also received the backward loop (LB) primer that bound to the amplicon loop between B1c and B2 sites that was not recognized by the respective OSD probe. The forward loop (LF) primers that overlapped the Tholoth and NB OSD binding regions were excluded. The LF primer was also initially excluded from the Lamb LAMP-OSD assay even though the amplicon loop that bound this loop primer was long enough to accommodate a nonoverlapping OSD reporter; this was done to fairly compare the amplification kinetics of all three assays in a five-primer format. In later versions of the assay with the Lamb primers, all six primers were included (designated 6-Lamb).

**TABLE 1:** LAMP primers and OSD probes

Primer or probe	Sequence	Reference
Tholoth-F3	TGCTTCAGTCAGCTGATG	38
Tholoth-B3	TTAAATTGTCATCTTCGTCCTT	38
Tholoth-FIP	TCAGTACTAGTGCCTGTGCCCACAATCGTTTTTAAACGGGT	38
Tholoth-BIP	TCGTATACAGGGCTTTTGACATCTATCTTGGAAGCGACAACAA	38
Tholoth-LF	CTGCACTTACACCGCAA	38
Tholoth-LB	GTAGCTGGTTTTGCTAAATTCC	38
Tholoth-LB-Bio	/5Biosg/GTAGCTGGTTTTGCTAAATTCC	This study
Tholoth-OSD-FAM	/56-FAM/ACAGGTGTAAGTGCAGCCCGTCTTACACCGTGC/3InvdT/	This study
Tholoth-OSD-Q	GACGGGCTGCACTTACACCTGT/3IABkFQ/	This study
NB-F3	ACCGAAGAGCTACCAGACG	39
NB-B3	TGCAGCATTGTTAGCAGGAT	39
NB-FIP	TCTGGCCCAGTTCCTAGGTAGTTCGTGGTGGTGACGGTAA	39
NB-BIP	AGACGGCATCATATGGGTTGCACGGGTGCCAATGTGATCT	39
NB-BIP-Bio	/5Biosg/AGACGGCATCATATGGGTTGCACGGGTGCCAATGTGATCT	This study
NB-FIP-Bio	/5Biosg/TCTGGCCCAGTTCCTAGGTAGTTCGTGGTGGTGACGGTAA	This study
NB-LF	CCATCTTGGACTGAGATCTTTCATT	39
NB-LB	ACTGAGGGAGCCTTGAATACA	39
NB-LB-Fam	/56-FAM/ACTGAGGGAGCCTTGAATACA	This study
NB-OSD-FAM	/56-FAM/CCGAATGAAAGATCTCAGTCCAAGATGGTATTTCT/3InvdT/	This study
NB-OSD-Q	TCTTGGACTGAGATCTTTCATTCGG/3IABkFQ/	This study
Lamb-F3	TCCAGATGAGGATGAAGAAGA	40
Lamb-B3	AGTCTGAACAACTGGTGTAAG	40
Lamb-FIP	AGAGCAGCAGAAGTGGCACAGGTGATTGTGAAGAAGAAGAG	40
Lamb-BIP	TCAACCTGAAGAAGAGCAAGAACTGATTGTCCTCACTGCC	40
Lamb-LF	CTCATATTGAGTTGATGGCTCA	40
Lamb-LB	ACAAACTGTTGGTCAACAAGAC	40
Lamb-LB-Bio	/5Biosg/ACAAACTGTTGGTCAACAAGAC	This study
Lamb-OSD-FAM	GTATGGTACTGAAGATGATTACCAAGGTAAACCTTTGGAATTTGGAC/36-FAM/	This study
Lamb-OSD-Q	/5IABkFQ/GTCCAAATTCCAAAGGTTTACCTTGGTAATCATCTC/3InvdT/	This study
NB-AND-OSD.long	AGAAATACCATCTTGGACTGAGATCTTTCATTCGGCTTCAATATATCCTATAAATCACAGTATCTAAGCCGTGTAAAGAGAACATCC/3InvdT/	This study
NB-AND-OSD.short	CTGTCTCGTCTGATCATCGTCAGAGCTGTAACTCAATCAATTATATATTATATCCGAATGAAAGATCTCAGTCCAAGA/3InvdT/	This study
Lamb-AND-OSD.long	GTATGGTACTGAAGATGATTACCAAGGTAAACCTTTGGAATTTGGCTTCAATATATCCTATAAATCACAGGATGTTCTCTTTACACGGCTTAGATAC/3InvdT/	This study
Lamb-AND-OSD.short	CAGCTCTGACGATGATCAGACGAGACAGTAACTCAATCAATTATTTATTATTTCCAAATTCCAAAGGTTTACCTTGGTAATCATCTC/3InvdT/	This study

10.1128/mSphere.00911-20.3 FIG S1 LAMP primer and OSD probe binding sequences in the SARS-CoV-2 genome. Binding regions for primers and OSD probes used in 6-Lamb (A), NB (B), and Tholoth (C) LAMP-OSD assays are annotated on the SARS-CoV-2 genomic RNA sequence. Forward and reverse directions of the annotation arrows indicate sense (same as genomic RNA sequence) and antisense (reverse complement of genomic RNA sequence) nature of the primer and probe sequences. Outer primer F3 and B3 binding regions are shown in red, inner primer FIP (F1-F2) and BIP (B1-B2) binding regions are shown in blue, while loop primer (LF and LB) binding regions are shown in green. The fluorophore (FAM) and quencher (Q) labeled OSD strand binding regions are highlighted in pink. Download FIG S1, TIF file, 0.3 MB. Copyright © 2021 Bhadra et al. 2021 Bhadra et al. https://creativecommons.org/licenses/by/4.0/ This content is distributed under the terms of the Creative Commons Attribution 4.0 International license.

**FIG 2:**
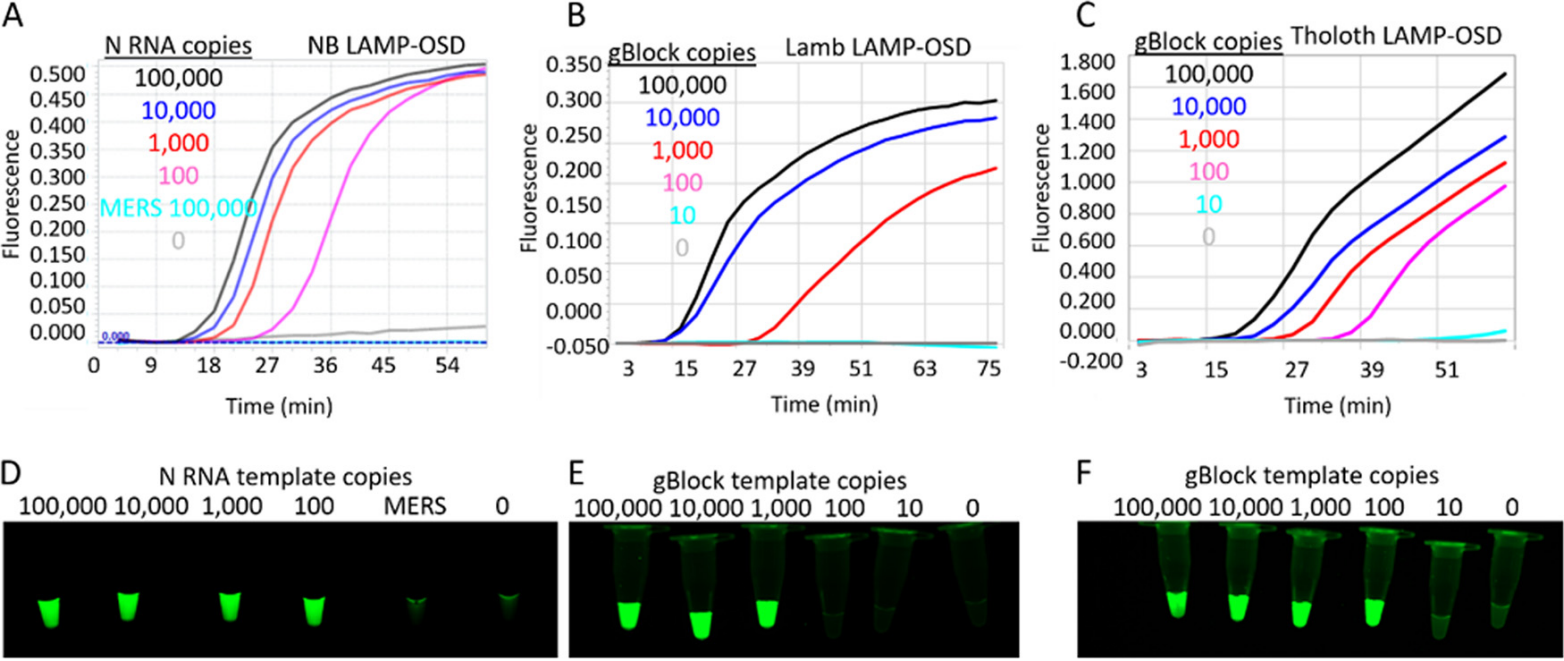
SARS-CoV-2 LAMP-OSD assays. OSD fluorescence measured in real time during LAMP amplification for NB (A), 5-Lamb (B), and Tholoth (C) LAMP-OSD assays are depicted as amplification curves. The presence or absence of OSD fluorescence visually observed at the assay endpoint after 90 min of amplification for NB (D), 5-Lamb (E), and Tholoth (F) LAMP-OSD assays is depicted by images of reaction tubes. NB LAMP-OSD assays were seeded with the indicated number of copies per reaction of SARS-CoV-2 N RNA or MERS-CoV N RNA or no templates. 5-Lamb and Tholoth LAMP-OSD assays were seeded with the indicated number of copies of gBlock DNA templates. Data are representative of three biological replicates.

**FIG 3:**
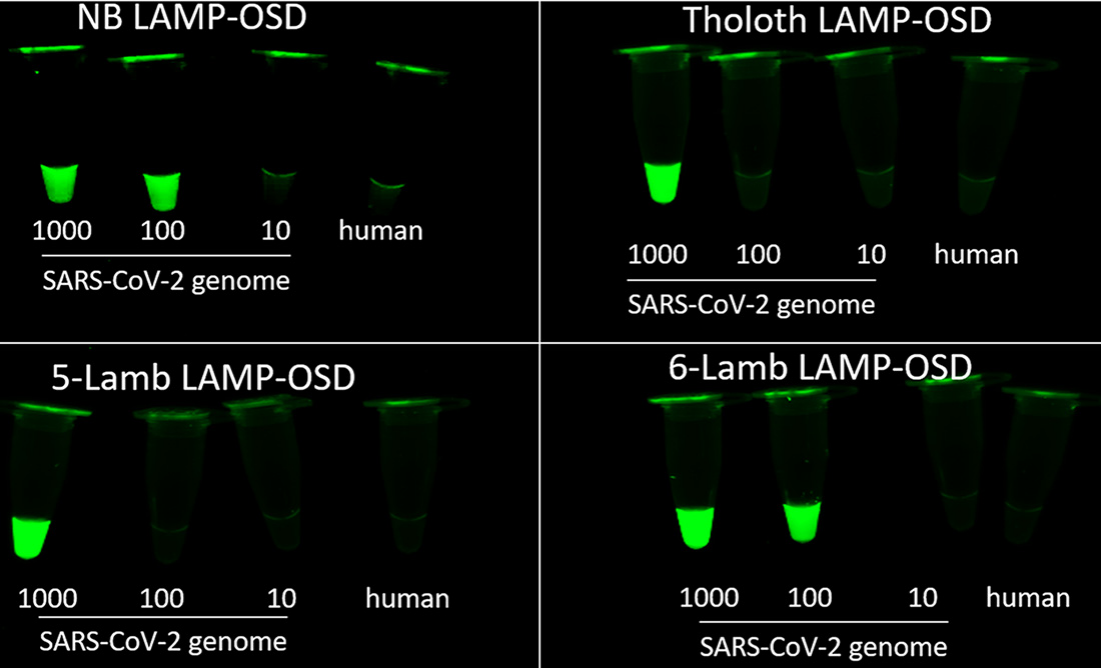
LAMP-OSD analysis of SARS-CoV-2 genomic RNA. The indicated numbers of copies of SARS-CoV-2 genomic RNA per reaction were analyzed using NB, Tholoth, and both 5-primer and 6-primer Lamb LAMP-OSD assays. Negative-control assays received 23 ng of human genomic DNA. Images of endpoint OSD fluorescence taken after 90 min of amplification are shown. Data are representative of four biological replicates.

For rapid prototyping, these LAMP-OSD assays were challenged with readily available *in vitro*-transcribed RNA or double-stranded DNA templates as surrogates for SARS-CoV-2 virions and viral genomic RNA. As shown in Fig. 2, in response to target templates, all three LAMP-OSD assays generated a strong OSD signal that could be both measured in real-time and observed visually at endpoint without interference from noise. No spurious signals were observed in response to RNA from other coronaviruses, such as Middle East respiratory syndrome coronavirus (MERS-CoV) (Fig. 2) or SARS-CoV Urbani (Fig. S3). We then tested the three LAMP-OSD assays using SARS-CoV-2 genomic RNA as the template. While the NB and Tholoth LAMP-OSD assays were performed using five primers (FIP, BIP, F3, B3, and LB), the Lamb LAMP-OSD assay was tested using either five primers (FIP, BIP, F3, B3, and LB) or six primers (FIP, BIP, F3, B3, LB, and LF). Amplification kinetics in representative assays were verified in real time (Fig. S3), and after 90 min of amplification at 65°C, the presence or absence of OSD fluorescence at endpoint was visually observed. As shown in Fig. 3, the presence of SARS-CoV-2 genomic RNA resulted in bright, easily detected fluorescence in all three LAMP-OSD assays. The six-primer version of Lamb LAMP-OSD could detect fewer genomic RNA copies than the five-primer version of the assay. In contrast, all assays showed no signal in the presence of only human genomic DNA. Differences in performance of various primer sets is likely due to the interplay of their propensities for spurious amplification (23), primer and foldback stabilities, amplicon lengths (region from F2 to B2), loop lengths, and amplicon GC contents.

10.1128/mSphere.00911-20.5 FIG S3 Specificity of SARS-CoV-2 LAMP-OSD assays. (A) Real-time OSD fluorescence accumulation in NB, 6-Lamb, and Tholoth LAMP-OSD assays seeded with 3,000 (black traces), 300 (red traces), or 0 (gray traces) copies of SARS-CoV-2 viral genomic RNA. Representative data from three biological replicates are depicted. (B) NB (tubes 2 and 5), 6-Lamb (tubes 3 and 6), and Tholoth (tubes 4 and 7) LAMP-OSD assays were seeded with either no templates (tubes 5, 6, and 7) or with 10,000 SARS-Urbani virions (tubes 2, 3, and 4). Multiplex NB + 6-Lamb LAMP-OSD assay seeded with 3,000 SARS-CoV-2 virions (tube 1) was used as a positive control. Images of endpoint OSD fluorescence taken after 90 min of amplification at 65°C are shown. Data are representative of two biological replicates. Download FIG S3, TIF file, 0.2 MB. Copyright © 2021 Bhadra et al. 2021 Bhadra et al. https://creativecommons.org/licenses/by/4.0/ This content is distributed under the terms of the Creative Commons Attribution 4.0 International license.

### Multiplex LAMP-OSD assay for SARS-CoV-2.

Multiplex assays designed to detect multiple sequences from an organism are often employed to improve the accuracy of identification (24, 25). The CDC-recommended diagnostic protocol for SARS-CoV-2 includes RT-qPCR amplification of at least two different regions of the viral genome. In fact, a recent prepublication demonstrated a multiplex PCR approach to enhance efficiency of detecting SARS-CoV-2 at low copy numbers (26).

Having determined that the individual LAMP-OSD assays with NB, Tholoth, and Lamb primers could signal the presence of SARS-CoV-2 RNA, we sought to execute these assays in a multiplexed format to create internally redundant assays for SARS-CoV-2. We chose to multiplex the NB assay with either the 6-Lamb assay or the Tholoth assay because they target different viral genes: the N gene and the ORF1AB region, respectively. We first tested the ability of both NB and Tholoth primer sets to amplify their respective synthetic targets (*in vitro* RNA transcripts of N gene and ORF1AB gBlock DNA templates) in a multiplex assay format by assembling LAMP-OSD reaction mixtures containing equimolar amounts of both LAMP primer sets with either only one or both OSD probes.

When these multiplex assays were seeded with both types of target templates, both Tholoth and NB primer sets led to an increase in their respective OSD fluorescence that could be readily distinguished visually from that of assays lacking specific templates at amplification endpoint (Fig. S4). Multiplex assays containing both OSD probes demonstrated an additive effect, with the OSD signal being brighter than that of assays containing only one type of OSD. Similarly, both NB and 6-Lamb primer sets could also amplify their respective targets in a multiplex assay (Fig. S4).

10.1128/mSphere.00911-20.6 FIG S4 Multiplex LAMP-OSD assay for SARS-CoV-2. Tholoth and NB (A) or 6-Lamb and NB (B) LAMP-OSD assays were combined in a multiplex format and analyzed using either individual or both OSD probes. Images of endpoint OSD fluorescence taken after 90 min (A) or 60 min (B) of amplification of indicated viral genomic RNA (gRNA) templates are shown. Integrated densities and plot profiles of each assay tube measured using ImageJ are depicted. Data are representative of three biological replicates. Download FIG S4, TIF file, 0.4 MB. Copyright © 2021 Bhadra et al. 2021 Bhadra et al. https://creativecommons.org/licenses/by/4.0/ This content is distributed under the terms of the Creative Commons Attribution 4.0 International license.

**FIG 4:**
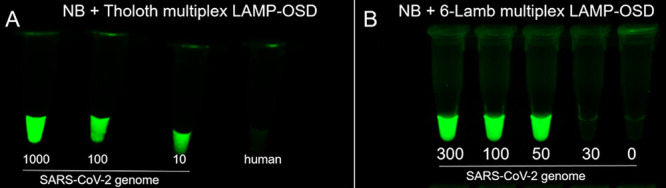
Multiplex LAMP-OSD analysis of SARS-CoV-2 genomic RNA. The indicated numbers of copies of SARS-CoV-2 genomic RNA per reaction were amplified at 65°C using NB plus Tholoth (NB + Tholoth) (A) or NB + 6-Lamb (B) multiplex LAMP-OSD assays for 90 min and 60 min, respectively. The control reaction received 23 ng of human genomic DNA. Images of OSD fluorescence captured after completion of amplification are shown. Data are representative of three biological replicates.

Having confirmed that both the primer sets are able to amplify their respective targets in one-pot multiplex reactions containing SARS-CoV-2 N gene and ORF1AB sequences, we tested the multiplex assays using full-length SARS-CoV-2 viral genomic RNA (Fig. 4). Visual observation of endpoint fluorescence revealed a bright signal in both types of multiplex assays containing only few tens of copies of SARS-CoV-2 genomic RNA. This sensitivity might be driven to a large extent by the NB primer set present in both multiplex assays, since it displays slightly faster amplification kinetics than that of both the Tholoth and Lamb primer sets (Fig. S3). Meanwhile reactions containing nonspecific human DNA remained dark (Fig. 4).

### Direct LAMP-OSD analysis of SARS-CoV-2 virion-spiked human saliva.

Given the low limits of detection we have observed, it is possible that LAMP-OSD might be used as part of diagnostics pipelines or in direct patient screening. However, for this, the reactions would need to operate under conditions commensurate with sample collection, especially in resource-poor settings. Collection of nasopharyngeal and oropharyngeal swab specimens causes considerable discomfort to patients and requires supplies in the form of sterile swabs and transport medium. Moreover, these samples are relatively difficult to self-collect. In contrast, saliva can be noninvasively collected simply by spitting in a sterile collection vessel, and it can be done just as easily in a clinic as at home. Furthermore, studies have shown that SARS-CoV-2 can be consistently detected in patient saliva, with median and mean viral loads of 3.3 × 10^6^ copies/ml and 3.8 × 10^5^ RNA copies/ml, respectively (27–29).

**FIG 5:**
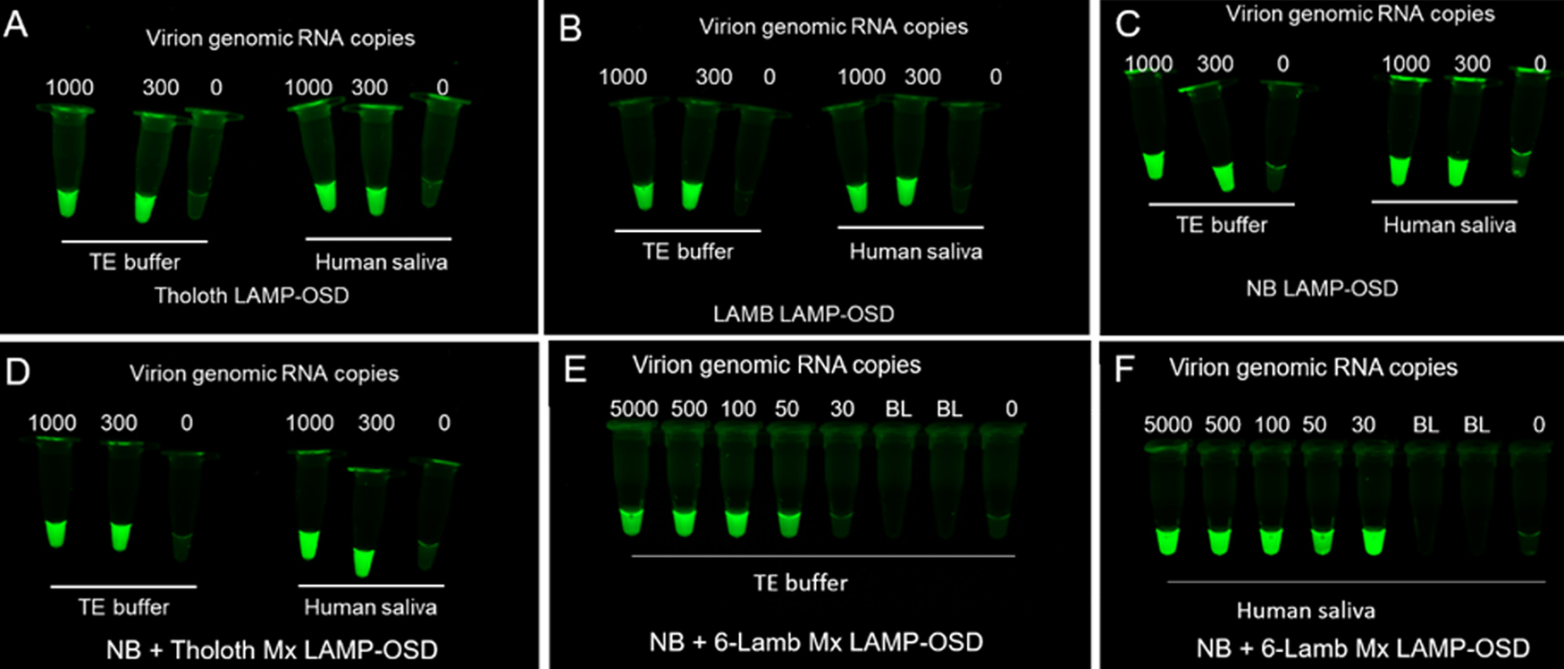
LAMP-OSD analysis of SARS-CoV-2 virions in the presence of human saliva. The indicated numbers of copies of virions per reaction were analyzed by individual or multiplex (Mx) LAMP-OSD assays in the presence of TE buffer or human saliva. Endpoint images of OSD fluorescence taken after 90 min of amplification are shown for Tholoth (A), 6-Lamb (B), and NB (C) individual LAMP-OSD assays and NB + Tholoth multiplex LAMP-OSD assays (D). Endpoint images of OSD fluorescence taken after 60 min of amplification are shown for NB + 6-Lamb multiplex assays (E and F). BL, blank tubes lacking any reaction mixtures or templates. Data are representative of four biological replicates.

We tested the direct sample analysis ability of individual and multiplex LAMP-OSD assays by seeding them with 3 μl of human saliva and different amounts of SARS-CoV-2 virions. As controls, duplicate LAMP-OSD reaction mixtures were seeded with virions suspended in 3 μl of Tris-EDTA (TE) buffer. Following 60 to 90 min of incubation at 65°C, endpoint observation of the presence or absence of OSD fluorescence revealed that all assays seeded with SARS-CoV-2 virions, whether in the presence of human saliva or TE buffer, were brightly fluorescent (Fig. 5). Even in the presence of saliva, LAMP-OSD could readily detect as few as 50 virions (in 3 μl saliva) per reaction (equivalent to ∼1.7 × 10^4^ SARS-CoV-2 virions/ml), an amount considerably lower than reported median and mean salivary SARS-CoV-2 viral loads (27, 29). In contrast, assays lacking specific templates remained noticeably darker than assays with specific templates. The faint fluorescence seen in some reaction mixtures containing saliva but no SARS-CoV-2 templates (for instance, in Fig. 5C) is due to sample autofluorescence and is readily distinguishable from the bright OSD fluorescence observed only in the presence of true amplicons (5, 17, 19). In several other direct sample analysis studies performed with various biological samples, such as environmental and wastewater and field-collected mosquitoes, the LAMP-OSD platform demonstrated accuracy on a par with gold standard methods such as qPCR (17, 19, 20, 30). These results suggest that LAMP-OSD assays might be used for direct analysis of human saliva samples in order to amplify and detect genetic signatures from SARS-CoV-2 virions. An accurate readout of a direct sample-to-fluorescence LAMP-OSD test can be readily achieved by comparing test fluorescence with a bright positive control, a dark “no-sample” negative control, and a reference reaction lacking LAMP primers, which would allow observation of sample autofluorescence. In a valid test, the negative control would be dark and the reference reaction would display minimal sample autofluorescence readily distinguishable from the bright positive control (Fig. S5). If signal brightness of the direct sample test is comparable to that of the positive control, the test would be considered positive for SARS-CoV-2. In contrast, if test fluorescence is as dim as the reference reaction, the test outcome would be negative (Fig. S5).

10.1128/mSphere.00911-20.7 FIG S5 Direct LAMP-OSD analysis of saliva samples preheated at 65°C. NB + 6-Lamb multiplex LAMP-OSD assays supplemented with 20 U of Superase·In RNase inhibitor (Thermo Fisher Scientific) (tubes 4, 5, 6, and 7) were seeded with the indicated number of copies of irradiated SARS-CoV-2 virions in the presence of 3 μl of human saliva that had been preheated at 65°C for 15 min. Positive-control (tube 1), reference (tube 2), and negative-control (tube 3) reaction mixtures comprising human *gapd* LAMP-OSD assays assembled with (tubes 1 and 3) or without (tube 2) primers were seeded with 3 μl of either water (tube 3) or saliva (tubes 1 and 2) preheated at 65°C for 15 min. Images of endpoint OSD fluorescence taken after 60 min of amplification at 65°C are shown. Data are representative of four biological replicates. Download FIG S5, TIF file, 0.1 MB. Copyright © 2021 Bhadra et al. 2021 Bhadra et al. https://creativecommons.org/licenses/by/4.0/ This content is distributed under the terms of the Creative Commons Attribution 4.0 International license.

### Logically integrated readout of multiplex LAMP-OSD using colorimetric lateral flow dipsticks.

To aid deployment under different local constraints, such as available instruments and reagents, human resources, and preferences, we sought to diversify assay platform options by adapting the LAMP-OSD assays for colorimetric readout by using lateral flow dipsticks. Since the OSD reporters are labeled with fluorescein, one of the simplest ways to transform LAMP-OSD signal into visible color accumulation is by incorporating biotinylated primers in the assay in order to generate LAMP amplicons that are dually labeled with biotin (via primer extension) and fluorescein (via OSD hybridization) (18). Such dually labeled amplicons can be readily detected using colorimetric lateral flow dipsticks, where they first bind to gold-labeled fluorescein-specific antibodies next to the sample application area and then are subsequently captured by biotin ligands immobilized at the test band, leading to generation of red color (Fig. S6A). In the absence of dually labeled analytes, gold particles accumulate only at the control band containing species-specific antibodies and no color develops at the test line.

10.1128/mSphere.00911-20.8 FIG S6 Colorimetric readout of individual LAMP-OSD assays using lateral flow dipsticks. (A) Schematic depicting method of colorimetric readout of LAMP-OSD assays using lateral flow dipsticks designed to detect analytes labeled with both biotin and fluorescein. AuNP, gold nanoparticle. LP-Biotin, biotin-labeled primer. (B) Cellphone images of colorimetric lateral flow readout of individual Tholoth, 6-Lamb, or NB LAMP-OSD assays seeded with the indicated number of copies of SARS-CoV-2 viral genomic RNA, followed by 60 min of amplification. Data are representative of three biological replicates. Download FIG S6, TIF file, 1 MB. Copyright © 2021 Bhadra et al. 2021 Bhadra et al. https://creativecommons.org/licenses/by/4.0/ This content is distributed under the terms of the Creative Commons Attribution 4.0 International license.

To enable colorimetric readout of Tholoth, Lamb, and NB LAMP-OSD assays on lateral flow dipsticks, we included biotinylated primers in each assay. In particular, for the Tholoth and 6-Lamb assays, the unlabeled LB primers were replaced with corresponding biotinylated primers, while the NB assays were appended with 0.4 μM additional FIP and BIP primers that were both labeled with biotin. Following 60 min of LAMP-OSD amplification, all three individual assays produced clearly distinguishable red colored test lines in the presence of few tens to hundreds of SARS-CoV-2 viral genomic RNA copies while producing no false signals in the absence of specific templates (Fig. S6B).

**FIG 6:**
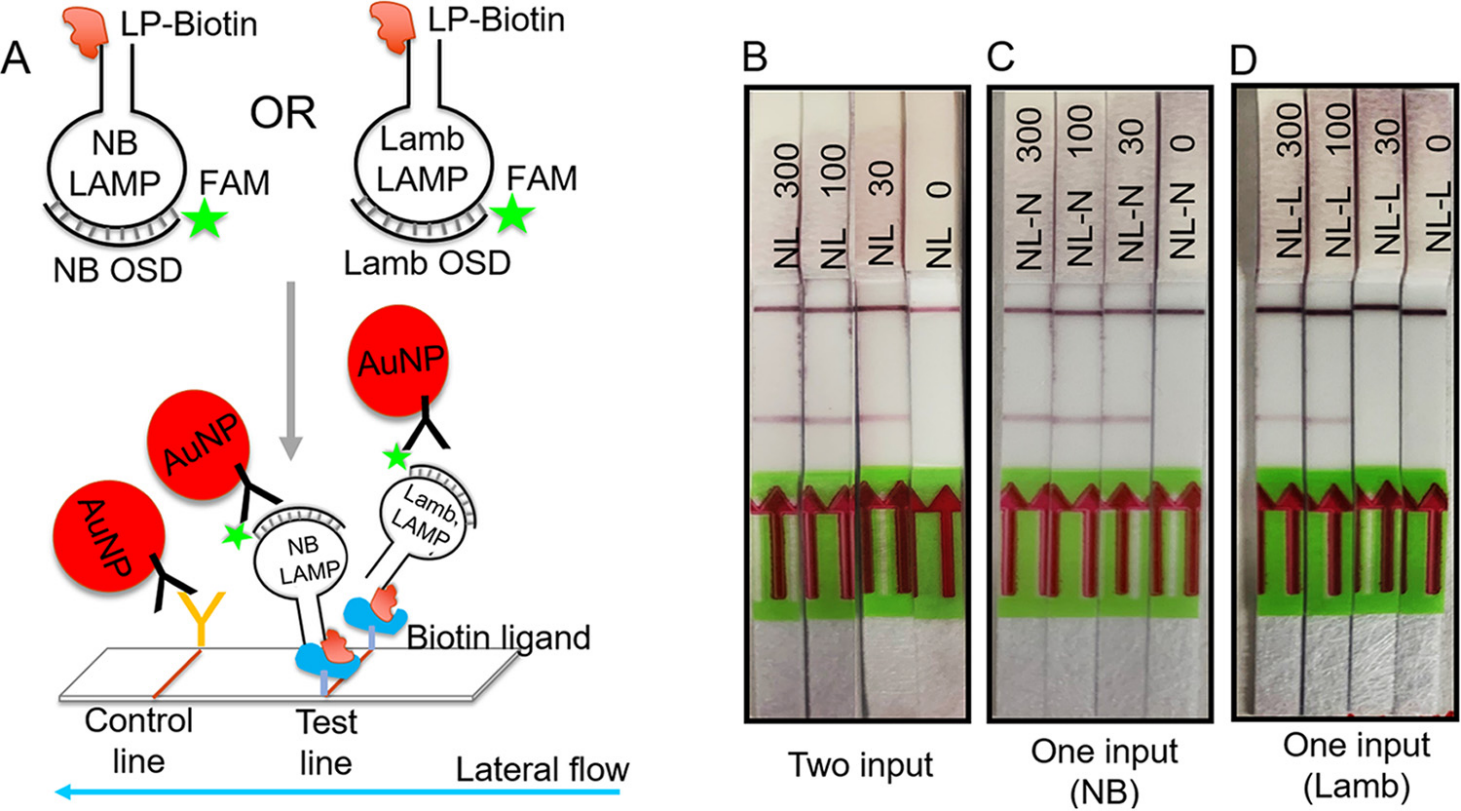
Colorimetric Boolean OR-gated readout of multiplex LAMP-OSD assays using lateral flow dipsticks. (A) Schematic depicting colorimetric Boolean OR logic-gated readout of multiplex LAMP-OSD assays using lateral flow dipsticks designed to detect analytes labeled with both biotin and fluorescein. AuNP, gold nanoparticle. LP-Biotin, biotin-labeled primer. (B, C, and D) Cellphone images of colorimetric lateral flow readout of NB and 6-Lamb (NL) multiplex LAMP-OSD assays seeded with the indicated numbers of copies of SARS-CoV-2 viral genomic RNA per reaction, followed by 60 min of amplification prior to analysis on lateral flow dipsticks. The multiplex assays contained either NB and 6-Lamb primer sets (NL) (B), NB and a nonspecific biotinylated LAMP primer set (NL-N) (C), or 6-Lamb and a nonspecific biotinylated LAMP primer set (NL-L) (D). Data are representative of three biological replicates.

We then reconfigured the NB and 6-Lamb multiplexed LAMP-OSD assay for execution of Boolean OR-gated lateral flow colorimetric readout. To confirm that the internally redundant assay generated a colorimetric signal when any one or both viral amplicons were produced, we executed the multiplex assay with either both NB and 6-Lamb primers or with only one type (NB or 6-Lamb) of the SARS-Cov-2-specific LAMP primer set to mimic the scenario in which one primer set fails to amplify its target. The omitted primer set was replaced with a nonspecific LAMP primer set containing all five primer types, including the same amount of biotinylated primers, in order to maintain similar concentrations of oligonucleotides and biotin. When tested with SARS-CoV-2 genomic RNA, the multiplexed NB and 6-Lamb LAMP-OSD assay generated distinct red colored test lines on lateral flow dipsticks upon amplification of one or both viral amplicons from a few tens of copies of viral templates without producing false signal (Fig. 6).

**FIG 7:**
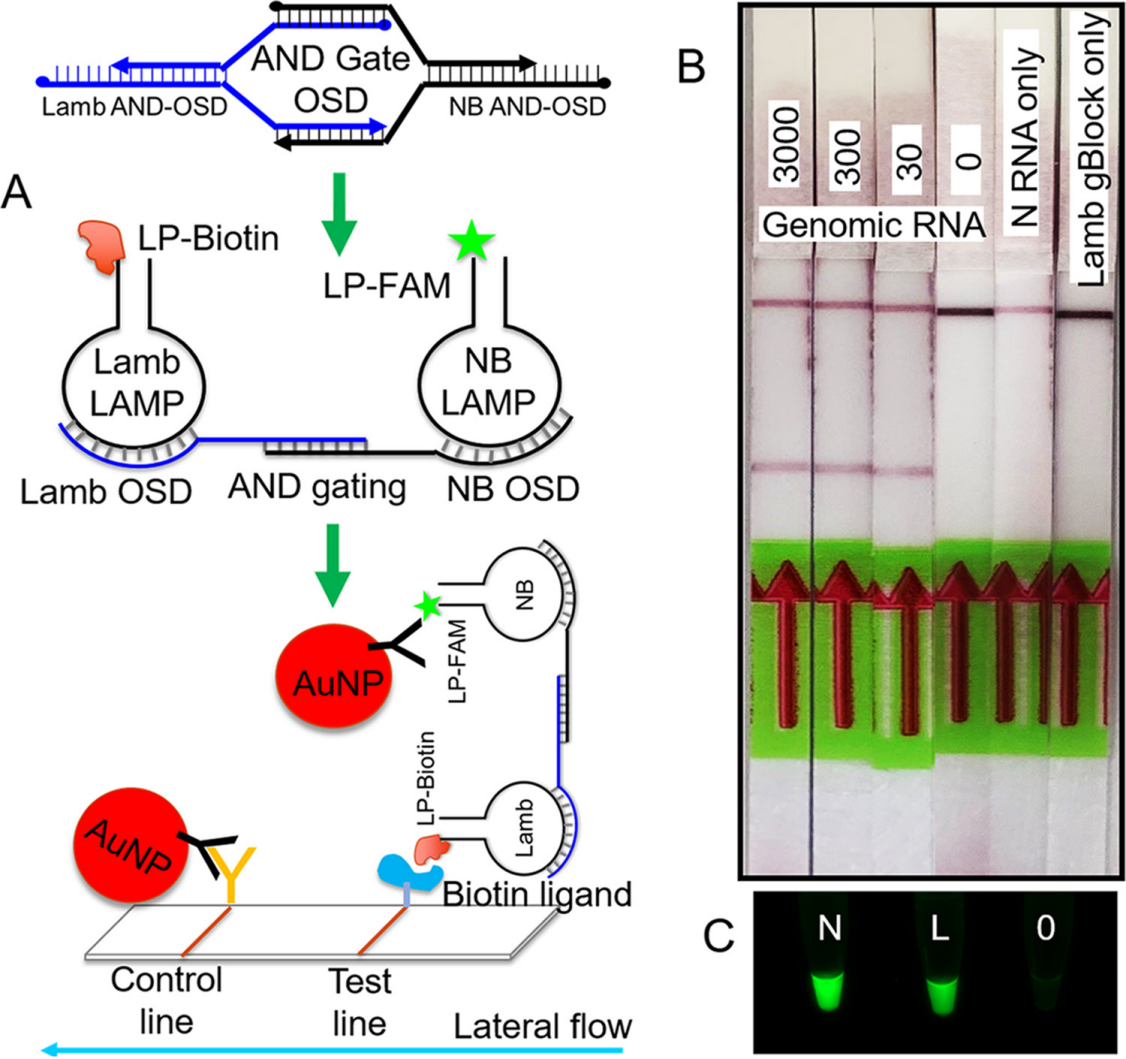
Colorimetric Boolean AND-gated readout of multiplex LAMP-OSD assays using lateral flow dipsticks. (A) Schematic depicting colorimetric Boolean AND logic-gated readout of multiplex LAMP-OSD assays using strand displacement gating probes (AND Gate OSD) and lateral flow dipsticks designed to detect analytes labeled with both biotin and fluorescein. AuNP, gold nanoparticle. LP-Biotin, biotin-labeled primer; LP-FAM, fluorescein-labeled primer. (B) Cellphone images of AND-gated colorimetric lateral flow readout of NB and 6-Lamb (NL) multiplex LAMP-OSD assays seeded with the indicated numbers of copies per reaction of SARS-CoV-2 viral genomic RNA (genomic RNA), 30,000 copies of only N gene armored RNA (N RNA only), or 30,000 copies of only Lamb-specific gBlock DNA (Lamb gBlock only), followed by 60 min of amplification prior to analysis on lateral flow dipsticks. (C) Multiplex LAMP-OSD assays comprising unlabeled primers and fluorogenic OSD reporters for both NB and 6-Lamb assays seeded with either no specific templates (0), 30,000 copies per reaction of only N gene armored RNA (N), or 30,000 copies of only Lamb-specific gBlock DNA (L). Endpoint images of OSD fluorescence taken after 60 min of amplification are shown. Data are representative of four biological replicates.

By querying the simultaneous presence of two or more target-specific amplicons, multiplex assays can also potentially enhance test accuracy by reducing false positives. In the simplest form, each amplicon in such multiplex assays is distinctly labeled for an independent readout. For instance, fluorophores with distinct emission spectra can be measured using a fluorimeter. Meanwhile, different small-molecule labels can enable amplicon capture and color development at two or more distinct test lines on specialized lateral flow devices. However, the added expense of multiple labels and the need for specialized devices might pose hurdles for widespread adoption. Therefore, we sought to develop an alternative readout mode that queries the copresence of multiple amplicons and generates a single visual signal only when all expected amplicons are present. To achieve this, we set up the multiplex assay using an NB primer set containing one fluorescein-labeled loop primer and a 6-Lamb primer set containing one biotinylated loop primer. When applied to fluorescein-biotin-specific lateral flow dipsticks, neither single-labeled amplicon by itself would generate a red color at the test line; the two labels must be conjoined to enable signaling. To form a physical bridge linking the two types of amplicons and hence labels that can then be detected on a lateral flow dipstick, we engineered a Boolean AND-gated OSD reporter module that would undergo sequence-specific strand displacement hybridization with both NB and Lamb LAMP amplicons (Fig. 7A). This complex would bind antifluorescein gold particles and would also be captured by the biotin ligand at the lateral flow test line, leading to color development. This approach minimizes the requirement for both differently labeled oligonucleotides and a specialized readout platform while ensuring the sequence specificity and logical computation of readout inherent in strand displacement reactions.

To test the AND-gated multiplex NB and 6-Lamb LAMP-OSD assay, we challenged it with purified genomic materials from infected cell cultures containing different numbers of copies of SARS-CoV-2 genomic RNA or with only the SARS-CoV-2 N gene armored RNA or the Lamb assay-specific gBlock template. Following 60 min of amplification at 65°C, the assays that had received even a few tens of copies of SARS-CoV-2 genomic RNA produced distinct red colored test lines on the lateral flow dipsticks (Fig. 7B). In contrast, assays without any specific templates produced only a red colored control line. Similarly, assays with only one type of viral template (N RNA or Lamb gBlock) also failed to produce a red colored test band despite producing individual amplicons (Fig. 7B and C). These results demonstrate the versatility of the LAMP-OSD platform and describe rapid reprogramming techniques for different testing modalities to meet varied/changing testing needs.

## DISCUSSION

In summary, we have demonstrated a facile way to rapidly configure LAMP assays for an accurate probe-based readout of SARS-CoV-2 by integrating OSD probes into individual and multiplex assays. These probes suppressed noise from spurious amplification by LAMP primers and thereby yielded target-specific signals. As a result, a few hundreds to a few tens of virion genomic RNA could be identified by individual or multiplex LAMP-OSD assays read by imaging probe fluorescence or by converting amplicon accumulation to color development on lateral flow dipsticks. In fact, the programmability of strand displacement probes allowed logical computation of the joint presence of viral amplicons on lateral flow devices. These results reinforce the fact that unlike many other fluorescence resonance energy transfer-based signal detection systems reported for LAMP, such as assimilating and DARQ probes (31, 32), strand displacement probes are versatile information processors that can be programmed to glean more sophisticated diagnostic information from LAMP amplicons than the mere presence or absence of a target sequence (17, 19). Consequently, integration of strand displacement, which was initially popularized as a mechanism for DNA computation (6), into LAMP has transformed this powerful nucleic acid amplification process into not only a more reliable method but also a more versatile and information-rich tool. In the future, it is likely that strand displacement probes will be one of the only means by which LAMP can be used in a highly multiplexed format to detect multiple pathogens in parallel. While there will be differences in cost-effectiveness of different assay modes for various application scenarios, ultimately, information programmability of strand displacement probes combined with their modular flexibility of signal transduction enables greater fungibility of readout platforms, which should in turn facilitate timely, cost-effective, and sustainable implementation that fits site-specific needs, available infrastructure, and human resources.

It is important to note that the enhanced sophistication of the LAMP-OSD platform does not compromise assay portability and ease of use. The SARS-CoV-2 LAMP-OSD assays can be executed in one-pot reactions assembled using individual reverse transcription LAMP reagents. An open reaction system with known components affords tremendous flexibility for fine-tuning the assays to meet local needs and constraints. In particular, to the extent that simply heating human saliva and adding it directly to LAMP-OSD reaction mixtures could lead to SARS-CoV-2 detection without spurious signals, this would engender robust sample-to-answer SARS-CoV-2 testing. Although our results are not yet clinically validated, they nonetheless suggest the feasibility of using LAMP-OSD for rapid and simple self-testing. Preheating the saliva by using just a heat block or water bath is one of the simplest and cost-effective ways to neutralize many amplification inhibitors, such as nucleases and proteases, while also providing the added benefit of reducing the viscosity of saliva and thereby making sample transfers easier and more uniform. In ongoing work using assays supplemented with RNase inhibitors, we have begun to demonstrate the feasibility of preheating saliva at only 65°C without compromising the accuracy of direct sample analysis via LAMP-OSD (see Fig. S5 in the supplemental material). With further saliva additives, such as chemical denaturants or protease inhibitors, it may become possible to eliminate sample pretreatment entirely. In addition, adaptation of SARS-CoV-2 LAMP-OSD assays to a low-temperature operation (33) may further reduce power requirements during point-of-care operation. We suggest that while LAMP-OSD may not often have the same sensitivity as “gold standard” RT-qPCR assays, the versatility of the LAMP-OSD assay, especially for resource-poor settings with limited infrastructure, might prove useful for screening for positives, which could then be followed up with more limited or difficult-to-execute RT-qPCR tests.

Beyond demonstrating high-surety assays for SARS-CoV-2, these results also serve as general guidance for rapid reconfiguration of LAMP assays into LAMP-OSD reactions that can be readily adapted for different readout modes, including multiplex readouts. Not only is the design of OSD probes straightforward, but their inclusion in LAMP assays requires minimum assay amendments. Furthermore, a one-pot operation and a visual readout eliminate procedural differences for the user while ensuring sequence specificity of signal similar to that afforded by TaqMan probes in qPCR.

In conclusion, the LAMP-OSD platform effectively combines simple one-tube operation with sophisticated nucleic acid sequence computation capacity. The user can anticipate robust and accurate answers from crude samples, based on modest technology requirements, features that are especially important for use under austere or resource-limited conditions. Moreover, deft platform adaptability, as demonstrated here by configuring SARS-CoV-2 assays for either colorimetric or logical probe-based readouts, should further promote synergies with local diagnostic needs and available infrastructure.

## MATERIALS AND METHODS

### Chemicals and reagents.

All chemicals were of analytical grade and were purchased from Sigma-Aldrich (St. Louis, MO, USA) unless otherwise indicated. All enzymes and related buffers were purchased from New England Biolabs (NEB, Ipswich, MA, USA) unless otherwise indicated. All oligonucleotides and gene blocks (Table 1) were obtained from Integrated DNA Technologies (IDT, Coralville, IA, USA). SARS-CoV-2 N gene synthetic transcript was a gift from the Schoggins laboratory at UT Southwestern Medical Center, Dallas, TX. SARS-CoV-2 genomic RNA and inactivated virions were obtained from the American Type Culture Collection, Manassas, VA, USA. SARS-CoV-2 N gene armored RNA was obtained from Asuragen, Austin, TX, USA. The HybriDetect universal lateral flow assay kit (Milenia Biotec, Gießen, Germany) for detection of biotin- and fluorescein-labeled analytes was purchased from TwistDx (Maidenhead, UK).

### OSD probe design.

We designed OSD probes (Table 1) for three recently described LAMP primer sets, here referred to as the Tholoth, Lamb (5-Lamb uses five primers, i.e., FIP, BIP, F3, B3, and LB, while 6-Lamb uses six primers, i.e., FIP, BIP, F3, B3, LB, and LF), and NB primers. The three primer sets target different regions in the ORF1AB and N genes of the SARS-CoV-2 genome. Fluorogenic OSD probes were designed for each of these primer sets using our previously described principles and the nucleic acid circuit design software NUPACK that is freely available at http://www.nupack.org/ (5, 34). Briefly, the target-derived loop regions between the F1 and F2 primer binding sites were chosen as OSD binding regions for each of the three LAMP primer sets (see Fig. S1 in the supplemental material). The long OSD strand was designed to be complementary to this loop region. Single-stranded 10- to 12-nucleotide-long toehold regions were designated on one end of this long strand, while a complementary short OSD strand was designed to hybridize to the remaining portion of the long strand. The long strand was labeled with a fluorescein moiety at the terminus not acting as the toehold. The short strand was labeled with a quencher, and all free 3′-OH ends were blocked with inverted dT to prevent extension by DNA polymerase.

Strand displacement probes for Boolean AND-gated reporting (AND-OSD) (Table 1) of Lamb and NB LAMP amplicon copresence on fluorescein-specific lateral flow dipsticks were designed by making the following modifications to the amplicon-specific OSD probes described above. The fluorescein moiety at the 3′ end of the Lamb OSD long strand was replaced with a 52-nucleotide-long random sequence engineered to act as a handle for hybridization to the NB AND-OSD long strand (Fig. 7A). Similarly, the Lamb AND-OSD short strand was extended with 53 random nucleotides at its 5′ end to act as a hybridization handle for the NB AND-OSD short strand. The NB AND-OSD probe was created by using the reverse complementary sequences of the long and short NB OSD strands such that the toehold was now situated at the 5′ end of the AND-OSD long strand. The 3′ end of the long strand and the 5′ end of the short strand of the NB AND-OSD probe were also extended with 52- and 53-nucleotide-long sequences, respectively. Both extensions included 28 base domains that were complementary to the Lamb AND-OSD long-and short-strand extensions, respectively (Fig. 7A). All free 3′-OH ends were blocked with inverted dT to prevent extension by DNA polymerase.

### Reverse transcription (RT) LAMP assay.

Individual LAMP assays were assembled in a total volume of 25 μl of 1× isothermal buffer [NEB; 20 mM Tris-HCl, 10 mM (NH_4_)_2_SO_4_, 50 mM KCl, 2 mM MgSO_4_, 0.1% Tween 20, pH 8.8, at 25°C]. The buffer was supplemented with 1.4 mM deoxynucleoside triphosphates (dNTPs), 0.4 M betaine, 6 mM additional MgSO_4_, 2.4 μM (each) FIP and BIP, 1.2 μM indicated loop primers, 0.6 μM (each) F3 and B3 primers, 16 U of *Bst* 2.0 DNA polymerase, and 7.5 U of WarmStart RTx reverse transcriptase. Amplicon accumulation was measured by adding OSD probes. First, Tholoth, Lamb, and NB OSD probes were prepared by annealing a 1 μM concentration of the fluorophore-labeled OSD strand with 2 μM, 3 μM, and 5 μM concentrations, respectively, of the quencher-labeled strand in 1× isothermal buffer. Annealing was performed by denaturing the oligonucleotide mix at 95°C for 1 min, followed by slow cooling at the rate of 0.1°C/s to 25°C. Excess annealed probes were stored at −20°C. Annealed Tholoth, Lamb, and NB OSD probes were added to their respective LAMP reaction mixtures at a 100 nM final concentration of the fluorophore-bearing strand.

Boolean OR logic processing fluorogenic multiplex RT-LAMP-OSD assays comprising both Tholoth and NB primers and probes were set up using the same conditions as described above, except that the total LAMP primer amounts were made up of equimolar amounts of Tholoth and NB primers. Boolean OR logic processing multiplex RT-LAMP-OSD assays comprising 6-Lamb and NB primers and probes were also set up using the same conditions as described above, with the exception that the total LAMP primer amounts were made up of equimolar amounts of 6-Lamb and NB primers supplemented with 0.2 μM (each) additional NB FIP and BIP primers.

Individual RT-LAMP-OSD assays for colorimetric lateral flow readout for Tholoth and 6-Lamb primer sets were set up as detailed above, except that the LB primers were replaced with equal amounts of the respective biotinylated LB primers. NB RT-LAMP-OSD assays for colorimetric lateral flow readout were set up as detailed above, except with inclusion of an additional 0.4 μM (each) biotinylated FIP and BIP primers. Boolean OR logic processing multiplex RT-LAMP-OSD assays for lateral flow readout were set up using NB and 6-Lamb primers and probes as detailed above, with the following exceptions: the 0.2 μM additional NB FIP and BIP were both biotinylated, and the NB and Lamb annealed OSD probes were used at final concentrations of 50 nM and 80 nM, respectively, of the fluorophore-labeled strand. Boolean AND logic processing multiplex RT-LAMP-OSD assays for lateral flow readout were set up in a total volume of 25 μl comprising 1× isothermal buffer, 1.4 mM dNTPs, 0.4 M betaine, 6 mM additional MgSO_4_, 1.2 μM (each) Lamb FIP and BIP, 1.2 μM (each) NB FIP and BIP, 0.6 μM biotinylated Lamb LB primer, 0.6 μM Lamb LF primer, 0.6 μM fluorescein-labeled NB LB primer, 0.3 μM (each) both NB and Lamb F3 and B3 primers, 16 U of *Bst* 2.0 DNA polymerase, 7.5 U of WarmStart RTx reverse transcriptase, and annealed AND-OSD probes at a final concentration of 100 nM. Annealed AND-OSD probes were assembled by mixing 1 μM (each) polyacrylamide gel-purified NB and Lamb AND-OSD short and long strands in 1× isothermal buffer, followed by 1 min of incubation at 95°C and slow cooling to 25°C at the rate of 0.1°C/s.

Templates were serially diluted in TE buffer (10 mM Tris-HCl, pH 7.5, 0.1 mM EDTA, pH 8.0) immediately prior to use, and 3 to 5 μl of these template preparations was included in each LAMP-OSD reaction mixture, achieving a total reaction volume of 25 μl. In some experiments, templates were introduced in LAMP reaction mixtures along with human saliva that had been heated at 95°C for 10 min. Templates used included zero to several hundred copies per reaction of synthetic double-stranded linear DNA gBlock (IDT, Coralville, IA, USA), *in vitro*-transcribed RNA, SARS-CoV-2 viral genomic RNA, inactivated SARS-CoV-2 virions, and inactivated SARS-CoV Urbani virions. Following addition of templates to RT-LAMP-OSD reagents, reaction mixtures were incubated at 65°C for the indicated duration.

Some LAMP-OSD reactions were analyzed in real time using a LightCycler 96 real-time PCR machine (Roche, Basel, Switzerland). Reaction mixtures were subjected to 30 cycles of two-step incubations: step 1, 150 s at 65°C; step 2, 30 s at 65°C. Fluorescence was measured in the 6-carboxyfluorescein (FAM) channel during step 2 of each cycle. LAMP-OSD assays intended for a visual yes/no readout of endpoint fluorescence were assembled in 0.2-ml optically clear thin-walled tubes with low autofluorescence (Axygen, Union City, CA, USA). Following the indicated duration of amplification at 65°C, endpoint fluorescence was imaged using either a cellphone camera and a blue light-emitting diode (LED) transilluminator or a Bio-Rad ChemiDoc camera (Bio-Rad Laboratories, Hercules, CA, USA).

A colorimetric lateral flow readout of fluorescein and biotin dually labeled RT-LAMP-OSD amplicons was performed using a HybriDetect universal lateral flow assay kit (Milenia Biotec, Gießen, Germany) for detection of biotin- and fluorescein-labeled analytes according to the manufacturer’s instructions. Briefly, following 1 h of amplification at 65°C, the entire 25 μl of an RT-LAMP-OSD reaction mixture was mixed with an equal volume of HybriDetect assay buffer (Tris-buffered saline). A HybriDetect dipstick was then placed upright in this solution such that only a portion of the sample application pad was immersed in the liquid. Upward lateral flow of liquid was allowed to occur for 5 to 15 min at room temperature prior to imaging of the colorimetric results with a cellphone camera.

10.1128/mSphere.00911-20.1 TEXT S1 Human *gapd* LAMP-OSD assay. Download Text S1, DOCX file, 0.01 MB. Copyright © 2021 Bhadra et al. 2021 Bhadra et al. https://creativecommons.org/licenses/by/4.0/ This content is distributed under the terms of the Creative Commons Attribution 4.0 International license.
