# Supplementary material for: High-Surety Isothermal Amplification and Detection of SARS-CoV-2
Source: mSphere. 2021 May 19;6(3):e00911-20. doi: 10.1128/mSphere.00911-20 (PMC8265673; doi:10.1128/mSphere.00911-20)
Supplement: TABLE S1 [file msphere.00911-20-st001.docx]

| **Primer ID number** | **Reference** | **Preprint Date** | **Gene Target** | **Temp (°C)** | **Preprint validation** |
| --- | --- | --- | --- | --- | --- |
| 1 (Tholoth) | *^1^* | 2020.02.16 | ORF1ab | 63 | Synthesized DNA |
| 2 (Lamb) | *^2^* | 2020.02.19 | ORF1a | 65 | Synthetic Patient Samples |
| 3 | *^3^* | 2020.02.20 | ORF1ab | 63 | Patient Samples |
| 4 | *^4^* | 2020.02.26 | ORF1a-A | 65 | Patient samples |
| 5 | *^4^* | 2020.02.26 | ORF1a-B | 65 | Patient samples |
| 6 | *^4^* | 2020.02.26 | ORF1a-C | 65 | Patient samples |
| 7 | *^4^* | 2020.02.26 | Gene N-A | 65 | Patient samples |
| 8 (NB) | *^4^* | 2020.02.26 | Gene N-B | 65 | Patient samples |
| 9 | *^5^* | 2020.03.02 | ORF1ab | 63 | Patient samples |
| 10 | *^5^* | 2020.03.02 | N | 63 | Patient samples |
| 11 | *^5^* | 2020.03.02 | E | 63 | Patient samples |

1. El-Tholoth M, Bau HH, Song J. A Single and Two-Stage, Closed-Tube, Molecular Test for the 2019 Novel Coronavirus (COVID-19) at Home, Clinic, and Points of Entry. ChemRxiv. 2020. Epub 2020/06/09. doi: 10.26434/chemrxiv.11860137.v1

10.26434/chemrxiv.11860137. PubMed PMID: 32511284; PubMed Central PMCID: PMC7251958.

2. Lamb LE, Bartolone SN, Ward E, Chancellor MB. Rapid detection of novel coronavirus/Severe Acute Respiratory Syndrome Coronavirus 2 (SARS-CoV-2) by reverse transcription-loop-mediated isothermal amplification. Plos One. 2020;15(6):e0234682. Epub 2020/06/13. doi: 10.1371/journal.pone.0234682. PubMed PMID: 32530929; PubMed Central PMCID: PMC7292379.

3. Yu L, Wu S, Hao X, Dong X, Mao L, Pelechano V, Chen WH, Yin X. Rapid Detection of COVID-19 Coronavirus Using a Reverse Transcriptional Loop-Mediated Isothermal Amplification (RT-LAMP) Diagnostic Platform. Clinical chemistry. 2020;66(7):975-7. Epub 2020/04/22. doi: 10.1093/clinchem/hvaa102. PubMed PMID: 32315390; PubMed Central PMCID: PMC7188121.

4. Zhang Y, Odiwuor N, Xiong J, Sun L, Nyaruaba RO, Wei H, Tanner NA. Rapid Molecular Detection of SARS-CoV-2 (COVID-19) Virus RNA Using Colorimetric LAMP. medRxiv. 2020:2020.02.26.20028373. doi: 10.1101/2020.02.26.20028373.

5. Yang W, Dang X, Wang Q, Xu M, Zhao Q, Zhou Y, Zhao H, Wang L, Xu Y, Wang J, Han S, Wang M, Pei F, Wang Y. Rapid Detection of SARS-CoV-2 Using Reverse transcription RT-LAMP method. medRxiv. 2020:2020.03.02.20030130. doi: 10.1101/2020.03.02.20030130.
