# Supplementary figures and images for: High-Surety Isothermal Amplification and Detection of SARS-CoV-2
Source: mSphere. 2021 May 19;6(3):e00911-20. doi: 10.1128/mSphere.00911-20 (PMC8265673; doi:10.1128/mSphere.00911-20)

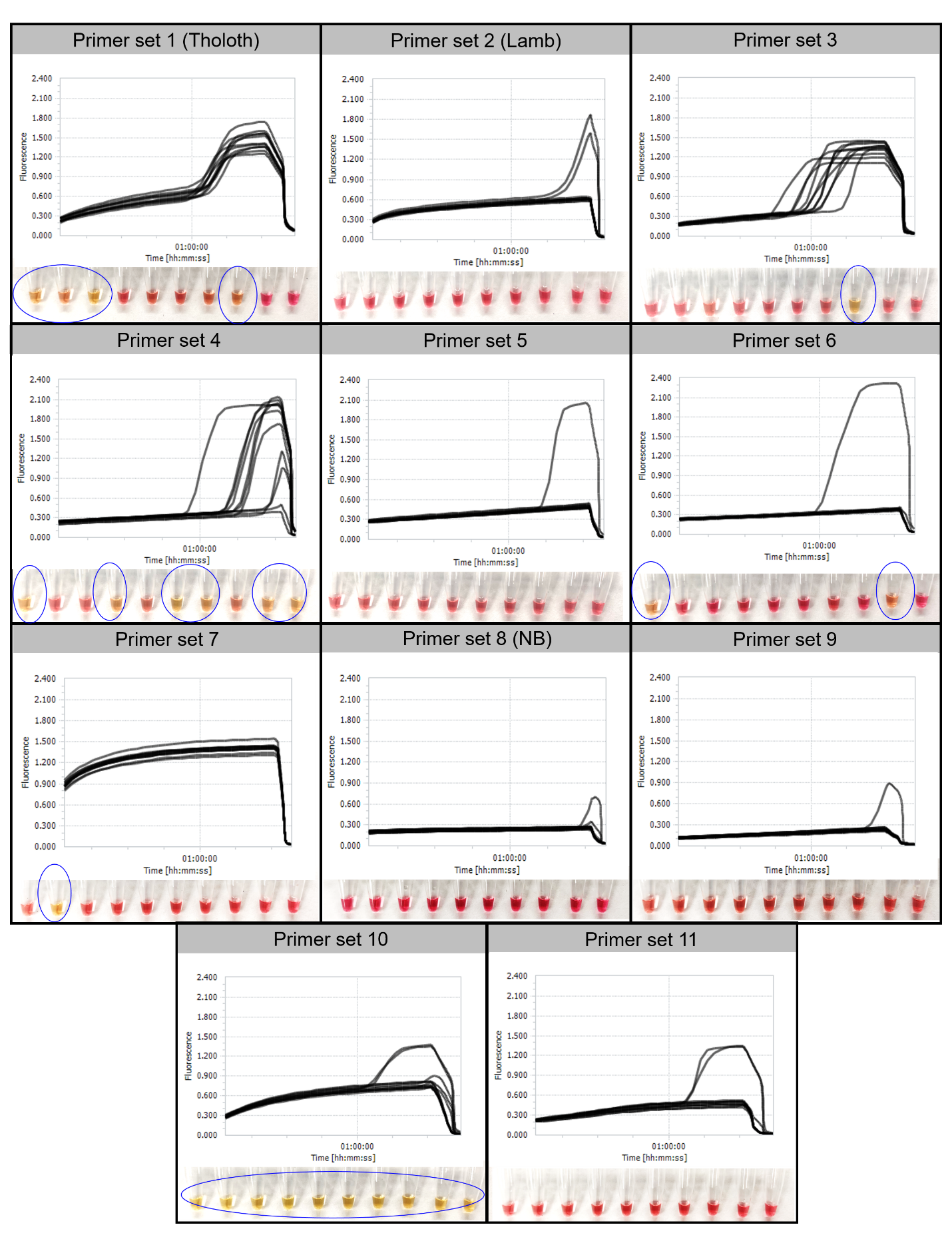

Supplement: FIG S2 [file msphere.00911-20-sf002.tif]

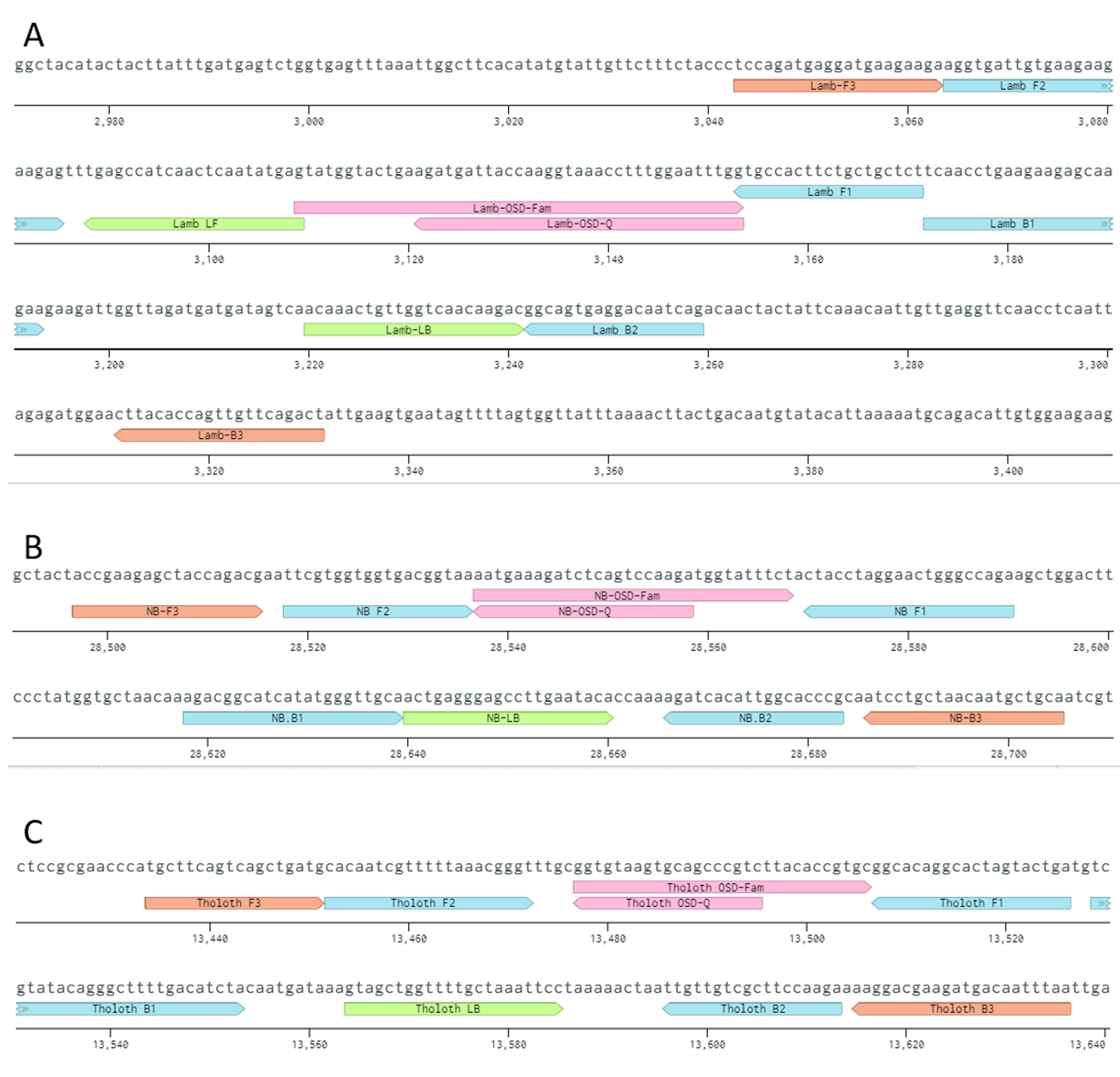

Supplement: FIG S1 [file msphere.00911-20-sf001.tif]

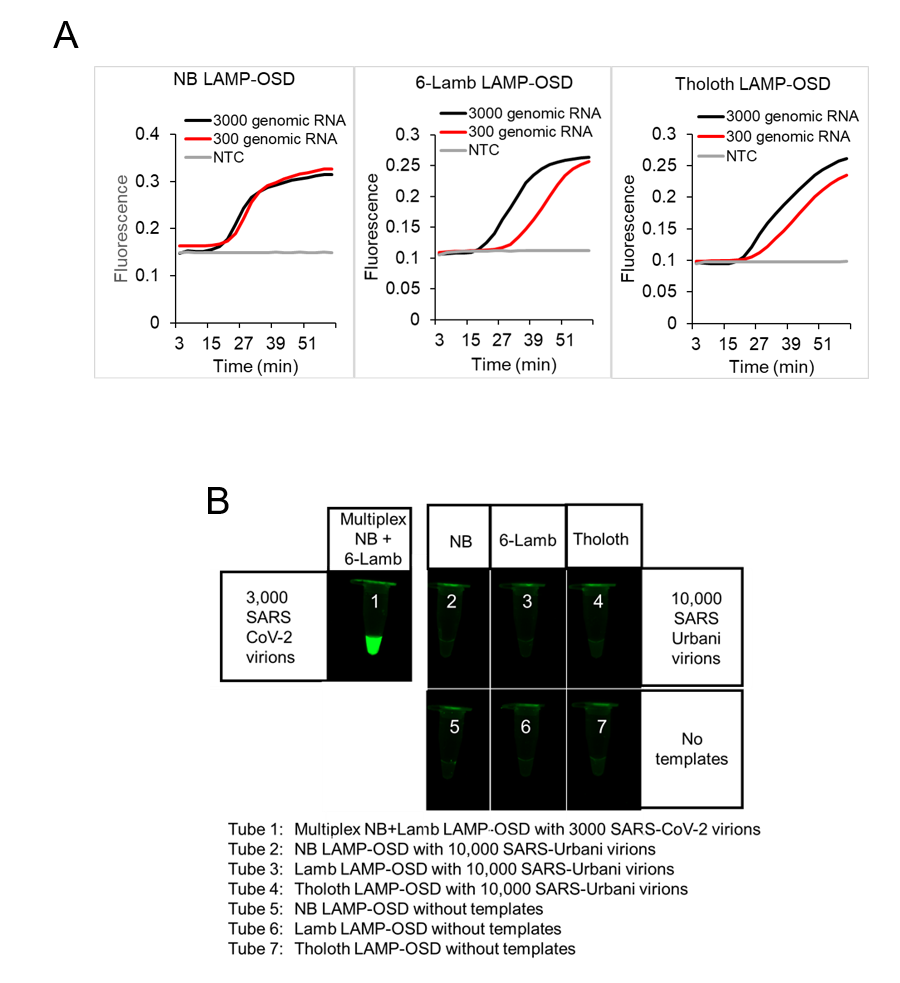

Supplement: FIG S3 [file msphere.00911-20-sf003.tif]

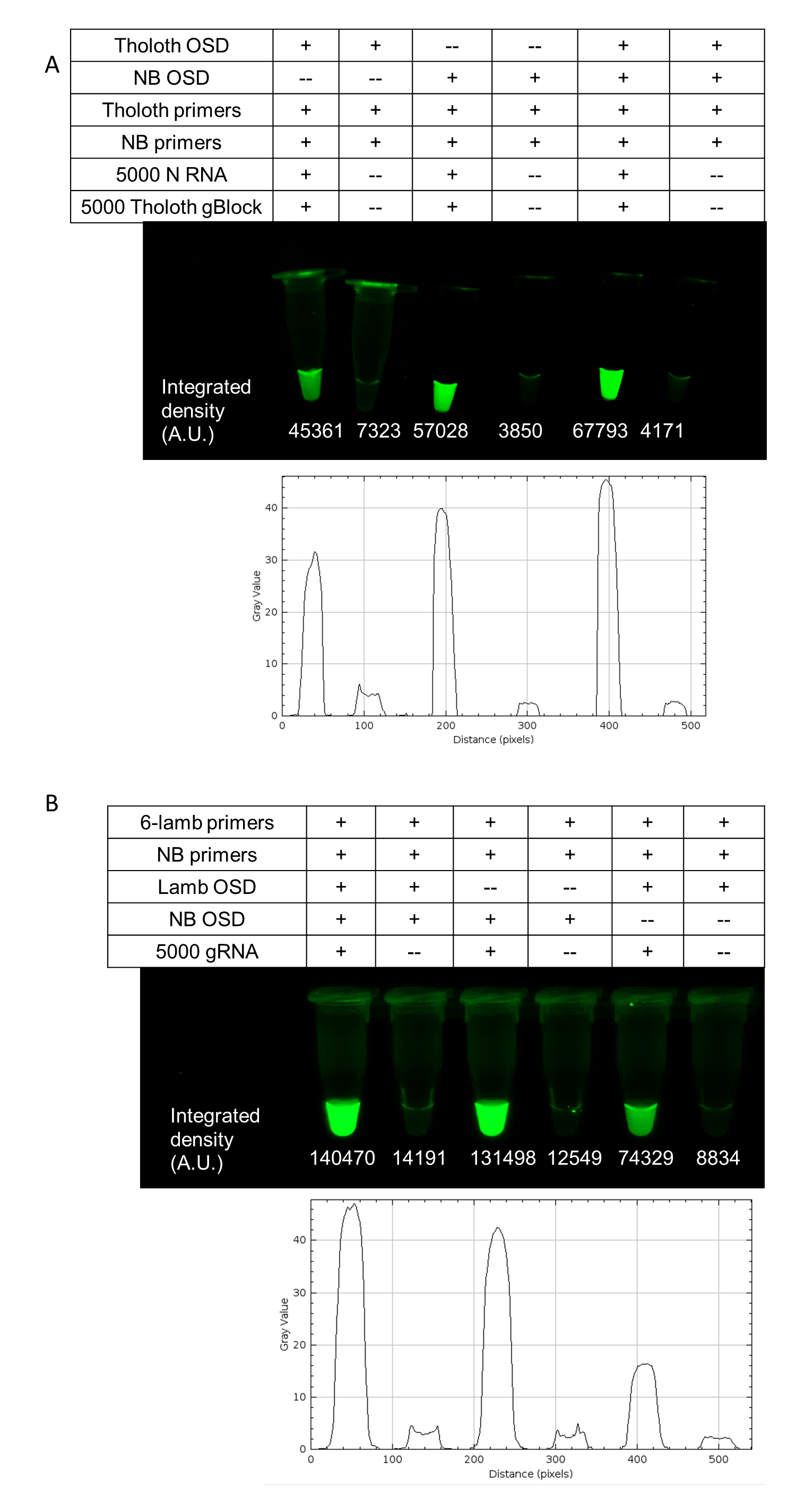

Supplement: FIG S4 [file msphere.00911-20-sf004.tif]

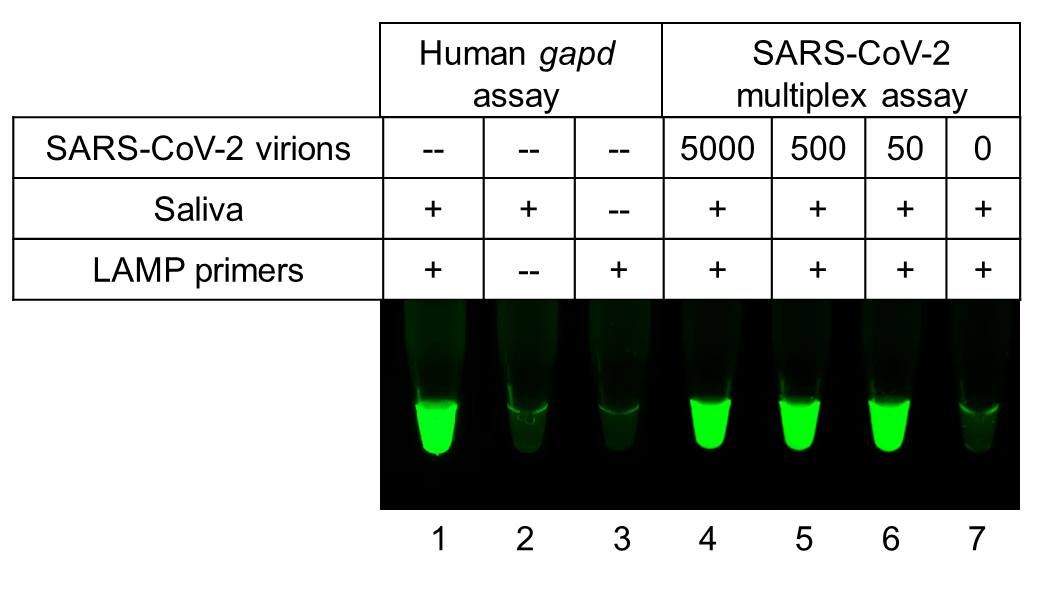

Supplement: FIG S5 [file msphere.00911-20-sf005.tif]

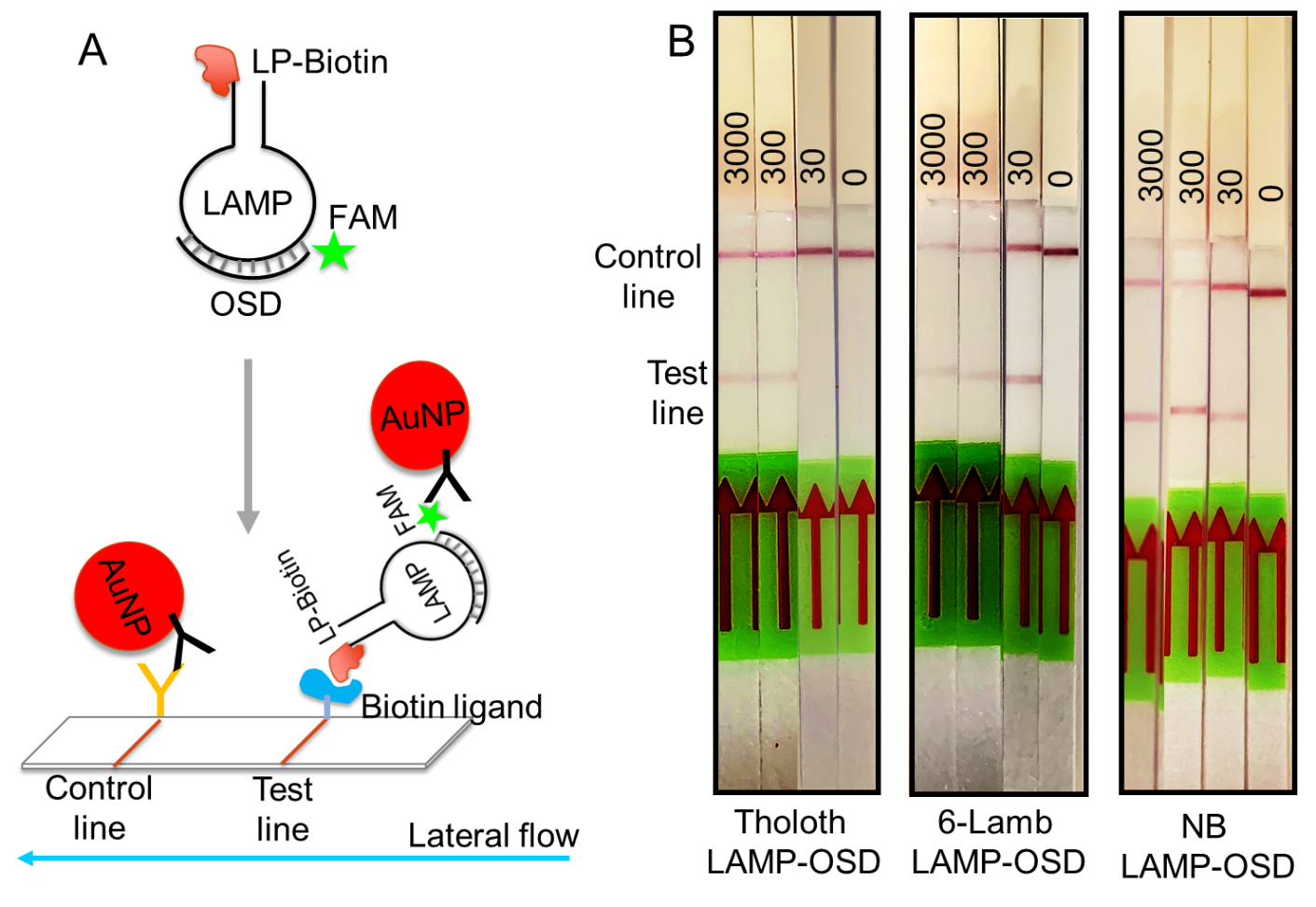

Supplement: FIG S6 [file msphere.00911-20-sf006.tif]
