# Supplementary material for: High-Surety Isothermal Amplification and Detection of SARS-CoV-2
Source: mSphere. 2021 May 19;6(3):e00911-20. doi: 10.1128/mSphere.00911-20 (PMC8265673; doi:10.1128/mSphere.00911-20)
Supplement: TEXT S1 [file msphere.00911-20-s0001.docx]

Human *gapd* LAMP-OSD assay

Human *gapd* LAMP-OSD assays were assembled in a total volume of 25 µL of 1X Isothermal buffer (NEB; 20 mM Tris-HCl, 10 mM (NH_4_)_2_SO_4_, 50 mM KCl, 2 mM MgSO_4_, 0.1% Tween 20, pH 8.8 at 25°C). The buffer was supplemented with 1.4 mM dNTPs, 0.4 M betaine, 6 mM additional MgSO_4_, 1.6 µM each of FIP and BIP, 0.8 µM of indicated loop primers, 0.4 µM each of F3 and B3 primers, 16 units of *Bst* 2.0 DNA polymerase, and 7.5 units of warmstart RTX reverse transcriptase. For some assays *gapd* LAMP primers were substituted with equal volume of TE buffer (10 mM Tris pH 7.5, 0.1 mM EDTA). Amplicon accumulation was measured by adding OSD probes. First, *gapd* OSD probes were prepared by annealing 1 µM of the fluorophore-labeled OSD strand with 2 µM of the quencher-labeled strand in 1X Isothermal buffer. Annealing was performed by denaturing the oligonucleotide mix at 95 °C for 1 min followed by slow cooling at the rate of 0.1 °C/s to 25 °C. Annealed OSD probes were added to *gapd* LAMP reactions at a final concentration of 100 nM of the fluorophore-bearing strand. Some 3 µL of either water or human saliva heated at 65 °C for 15 min were added to the assays, which were then incubated at 65 °C for 60 min prior to imaging endpoint OSD fluorescence.

| *Gapd* LAMP-OSD primer and probe sequences | |
| --- | --- |
| hGAPD.F3.1 | GCCACCCAGAAGACTGTG |
| hGAPD.B3.1 | TGGCAGGTTTTTCTAGACGG |
| hGAPD.FIP.1 | CGCCAGTAGAGGCAGGGATGAGGGAAACTGTGGCGTGAT |
| hGAPD.BIP.1 | GGTCATCCCTGAGCTGAACGGTCAGGTCCACCACTGACAC |
| hGAPD.LR.1 | TGTTCTGGAGAGCCCCGCGGCC |
| hGAPD.OSD.F | /56-FAM/CTCACTGGCATGGCCTTCCGTGTCCCCACTGCCAAC/3InvdT/ |
| hGAPD.OSD.Q | GGACACGGAAGGCCATGCCAGTGAG/3IABkFQ/ |
